# Local atomic structure studies of Zr_55_Cu_35_Al_10_ alloy around *T*_*g*_

**DOI:** 10.1038/s41598-023-36524-3

**Published:** 2023-06-06

**Authors:** Jingfeng Zhao, Yuhang Chen, Chucheng Shao, Jiang Liu, Genyu Zhu, Xuefeng Zhou

**Affiliations:** 1 School of Automotive Engineering, Changshu Institute of Technology, Changshu, 215500 People’s Republic of China; 2 Jiangsu Xinxunda Stainless Steel Products Co. LTD, Yancheng, 224000 People’s Republic of China

**Keywords:** Materials science, Mathematics and computing

## Abstract

As a result of examining the structure of Zr_55_Cu_35_Al_10_ alloy around the glass transition temperature (*T*_*g*_) using the classical molecular dynamics simulations, it was proven that the atomic bonds in the interconnecting zones (i-zones) became loose with the small amount of energy absorption, and it became free volumes easily when the temperature approached *T*_*g*_. Instead of i-zones, when clusters were largely separated by free volume networks, the solid amorphous structure was converted into supercooled liquid state, resulting in a sharp strength reduce and the great plasticity change from a limited plastic deformation to superplasticity.

## Introduction

It was thought that the atomic distribution of liquid at the temperature above the liquidus was random and uniform. However, with the development of various detection techniques, it was found that the atoms in the liquid presented short and medium range order. A new glassy metal with long-range disordered atomic arrangements-metallic glasses were often referred to as frozen metallic liquid. The topological model of completely disordered arrangement of atoms has been token for the atomic arrangement model of amorphous alloy for a long time after the discovery of amorphous alloy^1–5^. The free volume means the volume difference between the completely disordered arrangement of atoms and the ordered arrangement of crystals forms. The fraction of free volumes is often determined by the changes in the volume of amorphous alloys before and after crystallization. The concept of free volume is widely used to explain the physical and mechanical properties of metallic glasses^6–10^. However, researchers have found that the metallic glasses prepared at different cooling rates own different mechanical properties, and the metallic glassed prepared at different liquid temperatures have different thermal properties and present different crystallization process, which means that the atomic arrangement in the frozen metal liquid is not completely disordered, the frozen tissues under different temperatures should have corresponding short and medium range ordered structure, changing with the cooling rate^11,12^.

Metallic glasses show extremely high strength close to the theoretical value and an unusually large elastic strain because of their unique structural feature^13–15^. Compared with the aluminum, titanium, copper alloys and steel, the strength of Zr-based metallic glasses is more than twice that of Ti6Al4V and 17-4ss stainless steel. The linear elastic strain is ideally maintained to the yield point, which is more than twice that of ordinary alloys. Although metallic glasses have extremely high mechanical strength and physical properties, their macroscopic plasticity is very low. After the large linear elastic deformation and reaching the yield limit, the metal glass is deformed by highly localized shear band movement^16–19^. The thickness of the shear bands is only tens of nanometers. Although there is a large amount of deformation in the sheared zone, the metallic glass breaks only when several or only one shear zone is deformed, so the ductility deformation, which is much less than 1%, is often shown to have broken after reaching the yield limit^20–22^.

An understanding of the structure–property relation is the fundamental goal for the study of atomic structures. How to clarify the relationship between the structural models and physical and mechanical properties of glassy materials is important^23,24^. Differential scanning calorimetry (DSC) showed 0.79W/g energy absorption during glass transition. Yet, when the Zr-based metallic glass turns into the supercooled liquid state from the amorphous solid at room temperature, it only needs to absorb little energy, accompanied by a sharp strength reduce from 2000 to 70 MPa and the great plasticity change from a limited plastic deformation to superplasticity. How does the low energy absorption change the atomic structure of BMGs and brings liquid-like mechanical properties? The structure–property relations are related to not only the atomic packing geometrically but also the bonding properties among atoms, in which bonding length is one of the most important factors^29,30^.

Most recently, based on the studies of the atom-bonding lengths and atomic packings of local atomic structures using high-intensity neutron, high energy synchrotron diffraction technologies and molecular dynamics (MD) simulations, the atomic structure of the metallic glasses has been carefully characterized. The icosahedra, identified as the basic local structure in the metallic glasses, is widely studied in recent years. Celtek et al. presented that Al has a much greater effect on the formation of icosahedral short range order (SRO) than Ag and Zr_50_Cu_30_Al_20_ alloy has the best glass forming ability in the Zr_50_Cu_50−x_Al_x_ (x = 0, 10, 20, 30, 40, and 50) alloys by conducting MD simulations^25,26^. Zhang et al. reported that the distorted icosahedra ⟨0, 1, 10, 2⟩ has been identified as the basic SRO in many multicomponent metallic glasses using ab initio MD simulations^27^. Shimono et al.^28^ regarded a random network formed with Frank–Kasper clusters surrounded by icosahedral clusters as the basic medium-range order structure in metallic glasses by using MD simulations. In this research, the tight-bond cluster model for the metallic glasses has been adopted^29–37^. The tight‐bond cluster model mainly includes clusters, interconnecting-zones (i-zones) and free volumes, and it can be described as the clusters connected together by i-zones, and free volumes form among the clusters. The concept of the i-zones plays an important role in this model^29–31^. The nearest neighbor atoms are classified into cluster, i-zone, and free volume atomic bonds. In different alloy systems, the quantification of the cluster, the i-zone and the free volume bonding length can be defined by comparing the metallic glass structure with the corresponding crystallization structure obtained from the experiment in accordance with the radius of different elements in a particular system^32–37^. The tight-bond cluster model enables us to quantitatively characterize local atomic structures in amorphous alloys, contributing to explaining the phenomenon mentioned above.

## Computational method

In our work, classical molecular dynamics with embedded atom method (EAM) potentials proposed by Sheng et al. were used to investigate the structures of Zr_55_Cu_35_Al_10_ alloy around the glass transition temperature (*T*_*g*_)^5^. In the simulation, the cubic cell with initial arrangement of 16,000 atoms was set, and Zr, Cu and Al atoms were generated in random according to the composition of the alloy, and the initial velocity distribution obeyed the Maxwell Boltzmann distribution. The whole process was carried out in the three-dimensional periodic boundary conditions in the NPT ensemble with a Nose–Hoover thermostat for temperature control and a Nose–Hoover barostat for pressure control. The external pressure is set as zero. Firstly, the initial system temperature was set to 2.5 K, and then the temperature was raised to 2000 K to break the original structure and obtain a balanced liquid structure. After that, the system was cooled at a speed of 10^10^ K/s to 300 K to obtain the solid amorphous structure. The *T*_*g*_ in the simulation is identified at about 730 K through linearly fitting and extrapolating the two parts of the potential energy (PE) curve in the temperature range of 300–600 K and 900–1200 K, and the structures at the corresponding temperatures around *T*_*g*_ were obtained.

The tight-bond cluster model was used to describe the strength between atoms. The cluster bonds are formed with atoms with strong bonding; the free volume bonds have liquid-like loose bonding; the strength of the i-zone bonds fall in between. By comparing the pair distribution functions (PDFs) for the nearest atom pairs of the as-cast bulk metallic Zr_55_Cu_35_Al_10_ glass with its completely crystallized counterpart measured at the temperature of 15 K, cluster, i-zone and free volume bonds were defined^31–33^. The initial distances for the i-zone bonds are set at least 2.8% larger than their characteristic atomic radii, the initial distances for the free volume bonds are set at 9.6% greater than their characteristic atomic radii and the final positions for the free volume bonds are at the end of the first peaks in the corresponding partial PDFs. In addition, the bond formation energy for different atoms is calculated and expressed as follows:

1 $$ E_{p,B} = \frac{{(E_{p,\alpha } - E_{ref,\alpha } ) + (E_{p,\beta } - E_{ref,\beta } )}}{2}, $$

$E_{p,\alpha }$ presents the PE which can be obtained from the simulation. $E_{ref,\alpha }$ is the reference energy for the type $\alpha$ atom. The crystal PE (hcp Zr, fcc Cu and fcc Al) is used as the reference energy.

## Results and discussion

**Figure 1:**
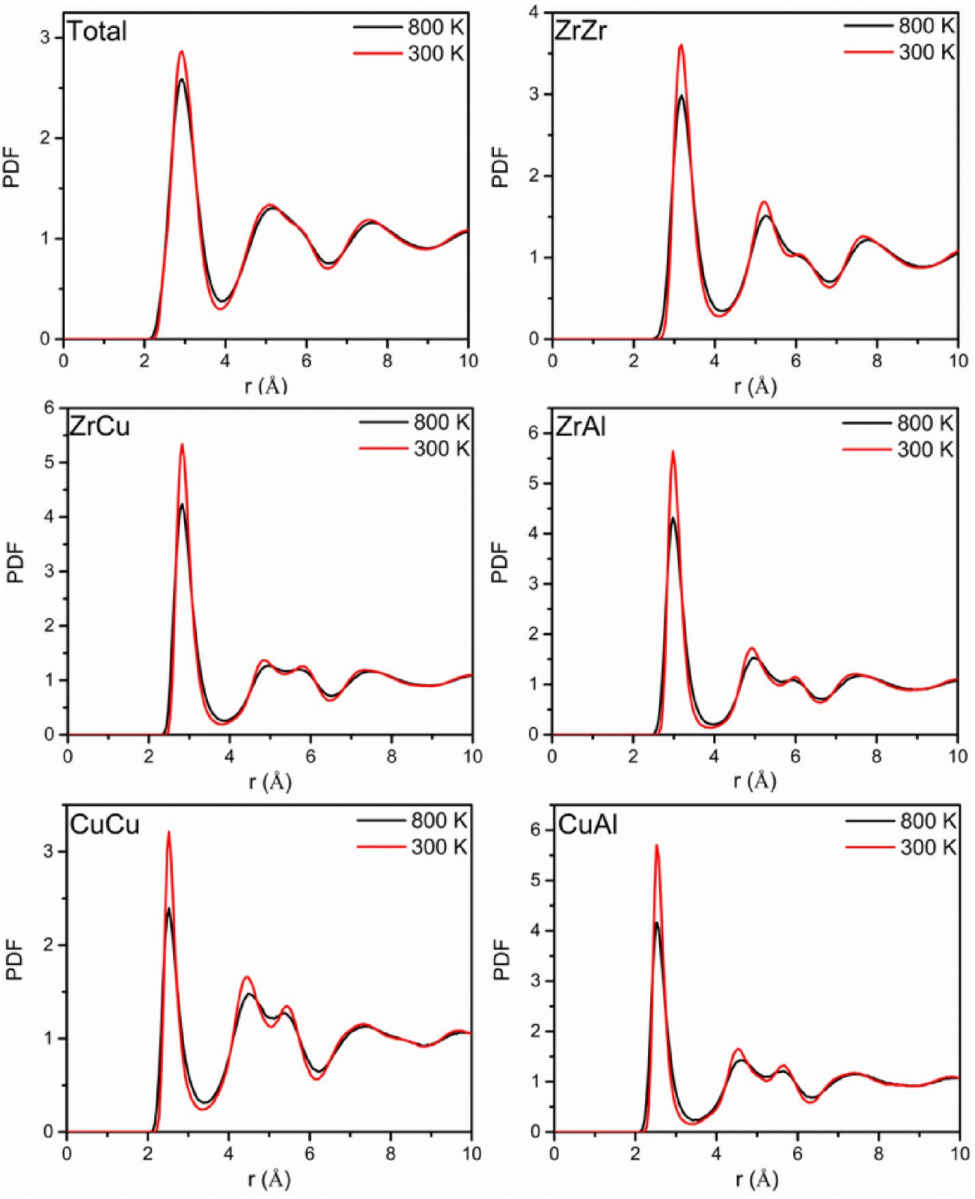
Comparison of total and partial pair distribution functions (PDFs) at 300 K and 800 K.

**Figure 2:**
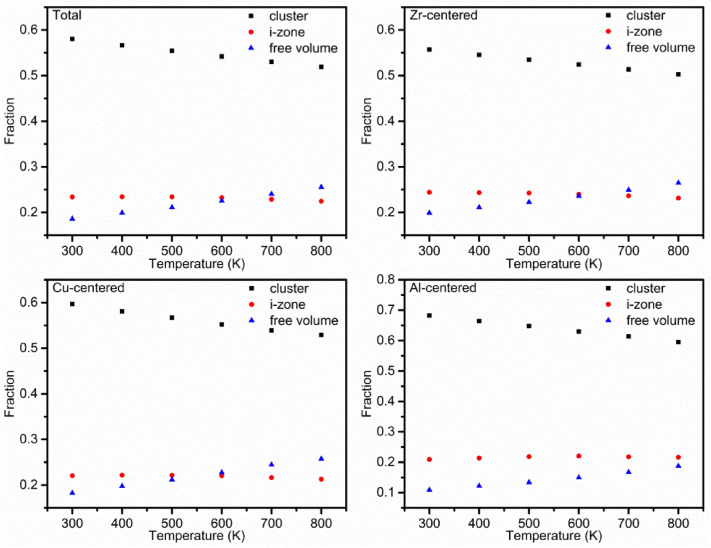
The variation of the fractions of the cluster, interconnecting zone (i-zone) and free volume atomic bonds from 300 to 800 K in the whole system and Zr, Cu and Al-centered atomic pairs.

Figure 1 shows the comparison of the total and partial PDFs at 300 K (the solid amorphous state) and 800 K (supercooled liquid state). It can be seen from the total and partial PDFs that the main difference exists in the first peak. The first peak strength increases significantly and the peak width decreases at 300 K, meaning that there are more atomic pairs with short bonding length at 300 K and it is more possible to form the atomic pairs with long bonding length near the first peak valley at 800 K. For the partial PDFs (ZrZr, ZrCu, ZrAl, CuCu and CuAl), the peak strength of the PDF curves for unlike atomic pairs (ZrCu, ZrAl, and CuAl) is much stronger than that of the like atomic pairs (ZrZr and ZrCu). The AlAl partial PDF is not presented here because of the low Al concentration and the poor statistics of calculating the AlAl partial PDF. It can be found from Fig. 2 that from 300 to 800 K, the fraction of the i-zone atomic bonds present slight decrease, the fraction of the free volume atomic bonds increases and the fraction of the cluster atomic bonds decreases significantly in the whole system and Zr, Cu and Al-centered atomic pairs, in accordance with difference in the first peak of the PDFs at 300 K and 800 K. For the Al-centered atomic bonds, the fraction of the cluster atomic bonds reaches about 0.68, which is much higher than that of the Zr-centered atomic bonds. The Al content is the least in Zr_55_Cu_35_Al_10_ alloy, but the fraction of Al cluster atomic bonds is the largest, indicating that the bonding between unlike atomic pairs Al–Zr or Al–Cu tend to be strong.

**Figure 3:**
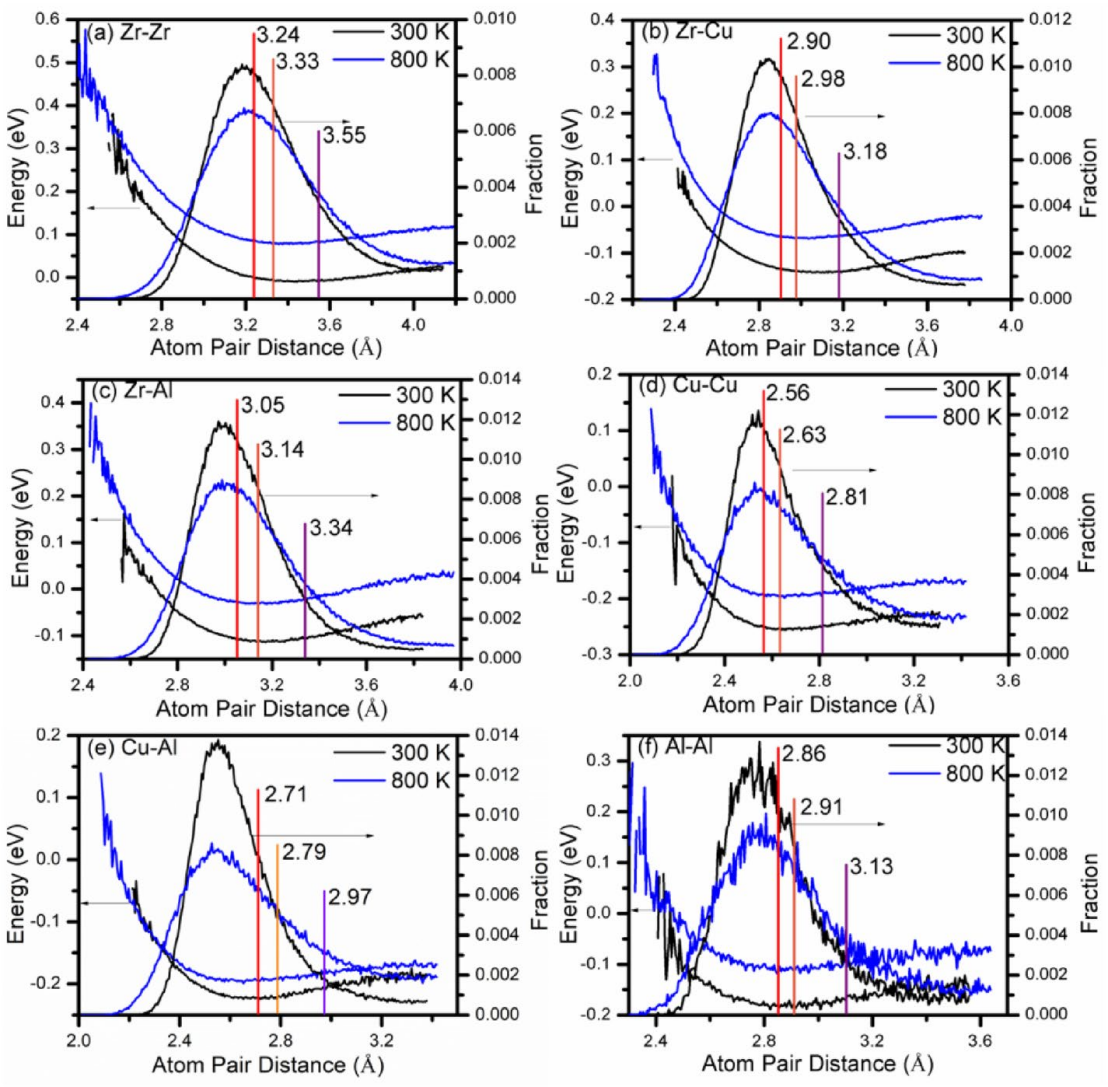
The change of the fraction and the energy of different atomic pairs (Zr–Zr, Zr–Cu, Zr–Al, Cu–Cu, Cu–Al and Al–Al) as a function of the atomic pair distance at 300 K and 800 K.

Figure 3 shows the fraction and energy distribution of different atomic pairs (Zr–Zr, Zr–Cu, Zr–Al, Cu–Cu, Cu–Al and Al–Al). In this figure, the characteristic position, the starting position of the i-zone atomic bonds and the starting position of the free volume atomic bonds for different atomic pairs are indicated by the red, orange and purple lines respectively. The atomic pairs between the orange line and purple line belong to i-zone atomic bonds, the atomic pairs to the left of the orange line belong to cluster atomic bonds and the atomic pairs to the right of the purple line belong to free volume atomic bonds. The fraction curves basically present Gaussian distribution, and the characteristic position is to the right of the peak position for different atomic pairs. By comparing the fraction curves of 300 K and 800 K, it is found that, the peak position basically unchanges while the peak intensity decreases significantly and the peak width increases, indicating the decrease of cluster atomic bonds and the increase of the free volume atomic bonds. The energy distribution curves are “U”-shaped, decrease firstly and then increase as the distance of the atomic pair increases at 300 K and 800 K. The low energy part is most between the orange line and purple line, corresponding to i-zone atomic bonds. It follows that i-zone is an important part of the structure and its existence can reduce the energy of the system, going a long way towards explaining the above phenomenon.

**Figure 4:**
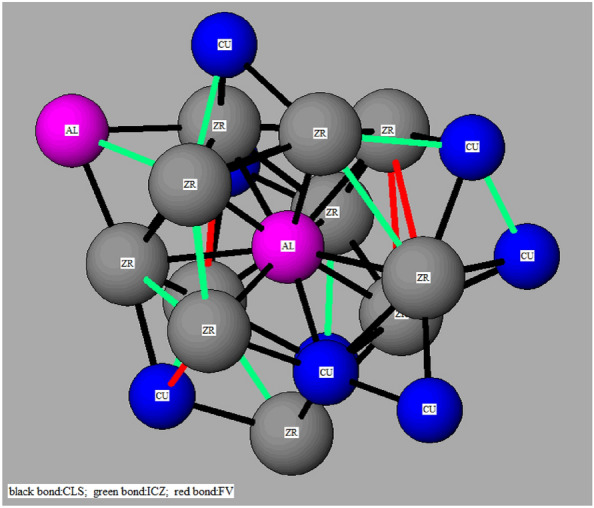
Al-centered cluster surrounded by i-zones and free volumes in local atomic structure. Cluster bonds is in black color, i-zones in green color, and free volume in red color.

The atom arrangements can be “physically” seen in the simulation. Figure 4 shows a cluster with i-zones and free volumes. The center of the cluster is Al, and its nearest surrounding atoms are Zr, which forms Zr–Al pairs, except one Cu–Al pair. The bonds shown by the green bars indicate i-zones, and with red bars are free volumes. The bonds in green bars, as well as those in the red bars are not isolated. They are associated with each other. Such it is easy to find that three bonds in green color have a shared atom, and the same of the red ones. It means that they exist as areas, which are able to form networks in the three dimensional space. The configuration of the i-zone and free volume networks should be very important for metallic glasses.

The structures from 300 to 800 K are picked up to make a thorough analysis. By tracking the atoms in the simulation from 300 to 800 K, it is found that all atoms only make a short distance migration leading to the expansion of the system with the shell atoms basically unchanged. When temperature increases from 300 to 800 K, the distance between atomic pairs increases, some of the cluster atomic bonds transform into i-zone atomic bonds, releasing energy, while some i-zone atomic bonds turn into free volume atomic bonds, absorbing energy. The absence of long distance migration of atoms and the transforms among the free volume, i-zone and cluster bonds should be the main reasons why the required energy of changing the structure from solid amorphous state at room temperature to the supercooled liquid is so small. However, the structure has undergone tremendous changes. When the temperature is 300 K, the proportion of cluster atomic bonds is very high, and the proportion of i-zone atomic bonds is much higher than that of free volume atomic bonds. When the temperature reaches 800 K, the fraction of cluster atomic bonds significantly decreases, the proportion of free volume atomic bonds has exceeded the proportion of i-zone atomic bonds. More free volumes are formed by consuming the clusters and i-zones. The clusters are mainly connected by free volumes instead of i-zones, the clusters could move and rotate more easily, forming a viscous liquid state. Therefore, there is a sharp decrease in strength and a sharp increase in plasticity when the temperature is above *T*_*g*_.

## Conclusions

In summary, the transformation of Zr-based alloys from the amorphous solid state to the supercooled liquid state requires very little energy absorption, yet the mechanical properties of Zr-based amorphous alloys have undergone great changes, including a sharp decrease in strength and a sharp increase in plasticity. The strength and plasticity change can be attributed to the variation of the distribution of the i-zones and free volumes around the clusters. When the temperature approaches *T*_*g*_, the i-zone atomic bonds become looser when gaining a small amount of energies, and easily to become to free volumes. When more clusters are surrounded by free volumes instead of the i-zones, the amorphous structure is then converted to the supercooled liquid structure, which results in the great change in the strength and plasticity since the clusters surrounded by the free volume could move smoothly.

## Data Availability

The datasets used and/or analysed during the current study available from the corresponding author on reasonable request.
